# Bayesian generalized linear mixed modeling of Tuberculosis using informative priors

**DOI:** 10.1371/journal.pone.0172580

**Published:** 2017-03-03

**Authors:** Oluwatobi Blessing Ojo, Siaka Lougue, Woldegebriel Assefa Woldegerima

**Affiliations:** 1 Mathematical Sciences, African Institute for Mathematical Sciences, AIMS-Cameroon, Limbe, Cameroon; 2 School of Mathematics, Statistics and Computer Sciences, University of Kwazulu-Natal, Durban, South Africa; 3 Department of Mathematics, Mekelle University, Mekelle, Ethiopia; Pennsylvania State University, UNITED STATES

## Abstract

TB is rated as one of the world’s deadliest diseases and South Africa ranks 9th out of the 22 countries with hardest hit of TB. Although many pieces of research have been carried out on this subject, this paper steps further by inculcating past knowledge into the model, using Bayesian approach with informative prior. Bayesian statistics approach is getting popular in data analyses. But, most applications of Bayesian inference technique are limited to situations of non-informative prior, where there is no solid external information about the distribution of the parameter of interest. The main aim of this study is to profile people living with TB in South Africa. In this paper, identical regression models are fitted for classical and Bayesian approach both with non-informative and informative prior, using South Africa General Household Survey (GHS) data for the year 2014. For the Bayesian model with informative prior, South Africa General Household Survey dataset for the year 2011 to 2013 are used to set up priors for the model 2014.

## Introduction

Tuberculosis (TB) has been rated as one of the world’s deadliest diseases [1]. It is caused by an obligate pathogenic bacterial specie in the family of *Mycobacteriaceae* called *Mycobacterium tuberculosis* (MTB) [2], and it is an airborne disease. Over a decade ago, TB disease was less predominant in Africa [3], but today, the occurrence of TB is greater in Sub-Sahara Africa. According to WHO 2014 world report, South Africa ranks 9^th^ out of 22 countries hit hardest by TB. The prevalence of TB is a real concern for authorities and also researchers to find better way to prevent it.

Past research on factors which influence TB epidemiology apart from its main cause by MTB, have shown that other conditions which affect the host cellular immunity, such as HIV disease, old age, diabetes, alcohol consumption, intense malnutrition and anti-tumour necrosis alpha factor (TNF-*α*) treatment, increase the risk of developing active TB. Other factors include contact with infected person, race, gender [4–6].

Over the past century, Bayesian statistics techniques have received various objections from the classical statisticians. This was mainly because of their intractabilities involved in calculating posterior distribution [7]. The posterior distribution is usually very complex and different from the usual distributions. But, with the discovery of Markov Chain Monte Carlo (MCMC) methods [8], Bayesian method started to gain momentum [7]. MCMC method is used in Bayesian inference to draw samples from the posterior distribution.

In Bayesian approach, the posterior distribution of the parameter is obtained by combining the prior distributions with the likelihood [7–9], while in classical statistics, only the likelihood is used as a basis for inference [9].

In Bayesian inference, the prior information can be informative or non-informative prior. A prior information is said to be informative prior if there is a solid external information about the distribution of the parameter of interest. While non-informative or vague prior are used in the case where no solid scientifically sound prior information is available about the parameter of interest. In this situation, a prior that will not have a significant influence on the posterior distribution is utilized.

The recent success of Bayesian techniques in data analysis is also imputable to the robustness and accuracy of the results produced by the approach. For this reason, this paper aim to provide knowledge on risk factors for TB in South Africa, using both the classical approach and Bayesian approach (with informative and non-informative priors). This is done using a generalized linear mixed model in both approaches.

## Materials and methods

### Data description

The dataset used for this paper is the South Africa General Household Survey yearly conducted by Statistics South Africa (Stats SA). This is a secondary data source collected and made freely available by Statistics South Africa (Stats SA) in their website (http://www.statssa.gov.za/). As the national institution providing statistics in South Africa, data from Stats SA are covered by ethical and scientific clearance and authorization. The main interest was on the GHS 2014 data. Bayesian and classical statistics models are fitted using the 2014 data. However, dataset for the years 2011, 2012 and 2013 are used to set up priors for Bayesian model with informative prior 2014. Classical and Bayesian analyses are implemented with the help of R [10] and WinBUGS [11] software, respectively.

GHS 2011 dataset has 93434 observations with 158 variables, 2012 dataset has 91859 observations with 156 variables, 2013 dataset has 93749 observations with 169 variables and 2014 dataset has 92459 observations with 183 variables.

The sampling technique used for the GHS is based on a Master Sample (MS) technique employed since 2008. The MS uses a two-stage sampling design. At the first stage, a stratified design with probability proportional to the size selection of Primary Sampling Units (PSUs) is used and at the second stage, a sampling of Dwelling Units (DUs) with systematic sampling is applied [12]. In 2009, an automated editing and imputation system was introduced by Stats SA for the GHS. This follows a standard set of rules that can be applied consistently over time.

The GHS’ target population is private households in all the nine provinces of South Africa and residents in workers’ hostels. Other collective living quarters like students’ hostels, hospitals, the old-age home, prison and military barracks were not cover by the survey [12].

The GHS 2014 sample is dominated by females. Among the 92459 people interviewed, 43476 (47%) are male and 48983 (53%) are females. Concerning the TB status of people interviewed, results show that 243 (0.3%) have TB, 91629 (99.1%) don’t have and 587 (0.6%) did not specify. In the analyses, the unspecified TB status were removed from the model. The racial distribution of the data indicates that height (8) people out of ten (10) interviewed (74287, 80%) are black Africans. GHS 2014 sample included 92459 people aged zero (0) years and above. Only people aged 15 years or above (64084, 69.3%) have been included in the model.

### Generalized linear mixed models

Generalized linear mixed models (GLMMs) are an extension of linear mixed models (LMM) and generalized linear models (GLM). GLMM is a generalization of linear mixed models (LMMs) to consider dependent variables from distributions other than normal such as count or binary responses. The extension of GLMs to GLMMs is done by the consideration of both fixed and random effects (mixed effects). While GLMs consider only fixed effects models, GLMMs incorporate additional random effects for situation dealing with longitudinal and complex structured (multivel) data. Fixed effects models assume that all observations are independent of each other, are not adequate for analysis correlated data structures such as clustered or multilevel data where observations are nested within groups.

The Generalized Linear Mixed Model is mathematically formulated as:

$$\psi \left(E\left(y|X,W,\phi \right)\right)=X^{{}^{\prime }}\beta +W^{{}^{\prime }}\phi +\epsilon ,$$ (1)

where *ψ* represents the link function, *Y* is an *N*_*obs*_ × 1 vector representing the dependent variable with *N*_*obs*_ been the total number of observations in the data and *X* is an *N*_*obs*_ × *N*_*c*_ matrix where each column represents one of the *N*_*c*_ covariates (level 1 predictors). *β* is an *N*_*c*_ × 1 vector representing the fixed-effects regression coefficients (coefficients linked to the predictors). *W* is an *N*_*obs*_ × *N*_*r*_ matrix including *N*_*r*_ random effects predictors (level 2 predictors); *ϕ* is a *N*_*r*_ × 1 vector of the random effects (coefficients linked to the random effect predictors); and *ϵ* is an *N*_*obs*_ × 1 vector of the residuals.

This study deals with a specific case of GLMM where the dependent variable is binary and two levels of analyses (individual and provincial levels) are considered. In such situation, the best model to use is the generalized linear mixed model with logistic link function, binary family with two levels of analyses. In this particular study, only the provinces’ IDs is considered at level two and no additional level two predictor is included in the model, therefore the *W* component is removed from the model. For a given observation *i* within a province *j*, the Generalized Linear Mixed Model (excluding the error term *ϵ*) then becomes:

$$\psi \left(E\left(y_{i}|X_{i},\phi_{j}\right)\right)=X_{i}^{{}^{\prime }}\beta +\phi_{j},$$ (2)

with *iϵ*1, …, *N*_*obs*_ and *jϵ*1, …, *N*_*p*_. This equation is the simplest mixed model called random-intercept model with a single random effect for each province *j* among all the *N*_*p*_ provinces. *ϕ*_*j*_ is the random effect (one for each province-level two unit) representing the influence of a province *j* on its within observations. Such influence is not captured by the observed covariates. It is important to include the random effects because the sampled individuals are supposed to be representative of the entire population in number and structure. The random effects are usually assumed to follow a normal distribution: $N(0,\sigma_{\phi }^{2})$ where $\sigma_{\phi }^{2}$ is the variance in the population distribution, and therefore the level of heterogeneity of observations in the data structure.

Furthermore, the model utilized a logistic link function, meaning:

$$\begin{array}{lll} \psi \left(E\left(y_{i}\right)\right) & = & logit\left(\Delta_{i}\right)\\ & = & log\left(\frac{\Delta_{i}}{1-\Delta_{i}}\right)\\ & = & log\left(\frac{Pr(y_{i}=1|X_{i},\phi_{j})}{1-Pr(y_{i}=1|X_{i},\phi_{j})}\right), \end{array}$$ (3)

where *E*(*y*_*i*_|*X*_*i*_, *ϕ*_*j*_) = Δ_*i*_ = *Pr*(*y*_*i*_ = 1|*X*_*i*_, *ϕ*_*j*_)

The final model is given as [13]:

$$\Delta_{i}=Pr\left(y_{i}=1|X_{i},\phi_{j}\right)=\frac{e^{X_{i}^{{}^{\prime }}\beta +\phi_{j}}}{1+e^{X_{i}^{{}^{\prime }}\beta +\phi_{j}}},$$ (4)

where *X*_*i*_ represents the predictors for an individual *i* in a province *j*, which can be quantitative (continuous), qualitative (categorical), or both (mixed). The response variable is denoted as *y*_*i*_, *β* is the vector of the fixed regression coefficients and *ϕ*_*j*_ is the random effect component [9].

The link function that maps the original value of *y*_*i*_ (0 or 1) to predictors is given as the log of the odds that an event occurs, this is known as the *logit* of Δ_*i*_ and is expressed as [13, 14]:

$$logit\left(\Delta_{i}\right)=\Omega_{i}=X_{i}^{{}^{\prime }}\beta +\phi_{j}.$$ (5)

In classical statistics, the estimated values of the logistic regression parameters that maximise the probability of obtaining the observed data [13] are usually computed using the method of maximum likelihood. The likelihood of *n* independent measurements, given vectors of parameter *θ* and explanatory variables *X*_*i*_ is expressed generally as [9]:

$$p\left(y|\theta ,X\right)=\prod_{i=1}^{N_{obs}}p\left(y_{i}|\theta ,X\right).$$ (6)

For binary logistic regression with response variable *y*_*i*_ = 0 or 1, the likelihood function is expressed as [9]:

$$p\left(y|\theta ,X\right)=\prod_{i=1}^{N_{obs}}\left\{\begin{array}{cc} {\text{logit}}^{-1}\left(\Omega_{i}\right) & \text{if}\;y_{i}=1\\ 1-{\text{logit}}^{-1}\left(\Omega_{i}\right) & \text{if}\;y_{i}=0, \end{array}\right.$$

this is equivalent to

$$\begin{array}{lll} p(y|\theta ,X) & = & \prod_{i=1}^{N_{obs}}{\left({\text{logit}}^{-1}\left(\Omega_{i}\right)\right)}^{y_{i}}{\left(1-{\text{logit}}^{-1}\left(\Omega_{i}\right)\right)}^{1-y_{i}}\\ & = & \prod_{i=1}^{N_{obs}}\Delta_{i}^{y_{i}}{\left(1-\Delta_{i}\right)}^{1-y_{i}}. \end{array}$$ (7)

To compute the estimate of *β* which maximises the likelihood function, we need to differentiate it with respect to *β*. To make this easier, we first take the natural logarithm (ln) of the likelihood function. That is:

$$\ln\left(p\left(y|\beta ,\phi_{j},X\right)\right)=\sum_{i=1}^{N_{obs}}\left[y_{i}\ln\left(\Delta_{i}\right)+\left(1-y_{i}\right)\ln\left(1-\Delta_{i}\right)\right].$$ (8)

Next is to take the partial derivatives of the log likelihood with respect to the parameters (*β*), set the equation to zero and solve to obtain those parameters which are candidate for maximum. Furthermore, it is sufficient to check if the second derivative is positively defined for those parameters.

The Wald test is applied to compute the confident interval of the parameters and it is given as [13]:

$$\hat{\beta_{k}}\pm z_{1-\frac{\alpha }{2}}\hat{SE}\left(\hat{\beta_{k}}\right).$$ (9)

Where $z_{1-\frac{\alpha }{2}}$ is the critical value of a standard normal distribution for a two sided test of size *α* and $\hat{SE}$ is the estimate of the standard error. *β*_*k*_ is fixed effect coefficient related to the *k*^*th*^ covariate.

The deviance statistic is used to test how well a model fits the data. It is a likelihood ratio statistic for comparing fitted model to the saturated model and is expressed as [13, 15]:

$$D=-2\ln\left[\frac{\left(\text{likelihood}\;\text{of}\;\text{the}\;\text{fitted}\;\text{model}\right)}{\left(\text{likelihood}\;\text{of}\;\text{the}\;\text{saturated}\;\text{model}\right)}\right].$$ (10)

Using the log likelihood equation given in Eq (8), we have:

$$\begin{array}{lll} D & = & -2\left[\sum_{i=1}^{N_{obs}}\left[y_{i}\text{ ln}\left(\Delta_{i}\right)+\left(1-y_{i}\right)\text{ ln}\left(1-\Delta_{i}\right)\right]-\sum_{i=1}^{N_{obs}}\left[y_{i}\text{ ln}\left(y_{i}\right)+\left(1-y_{i}\right)\text{ ln}\left(1-y_{i}\right)\right]\right],\\ & = & -2\sum_{i=1}^{N_{obs}}\left[y_{i}\text{ ln}\left(\frac{\Delta_{i}}{y_{i}}\right)+\left(1-y_{i}\right)\text{ ln}\left(\frac{1-\Delta_{i}}{1-y_{i}}\right)\right]. \end{array}$$

### Classical approach

Classical inference supposes that the model parameters are fixed, though they are unknown and that the data are random. It means that for a given parameter *θ*, the probability of the observed data *y* can be written as: *p*(*y*|*θ*) [7, 8].

For a logistic regression, the multilevel model for varying intercept is given as [9]:

$$Pr\left(y_{i}=1\right)=logit^{-1}\left(X_{i}^{{}^{\prime }}\beta +\phi_{j}\right)$$

with

$$\phi_{j}∼N\left(0,\sigma_{\phi }^{2}\right),$$

where *σ*_*ϕ*_ is the standard deviation of the unexplained group-level errors [9].

### Bayesian approach

Bayesian inference assumes that the data are fixed and considers all unknown parameters as random variables. If we consider a given parameter *θ* and a set of observed data, the Bayesian approach will be interested in the probability of the parameter *θ* given the set of data available *y*, mathematically this can be written as: *p*(*θ*|*y*) [7, 8]. The main interest is in computing the posterior distribution of the unknown parameter *θ* given the observed data *y*. This is obtained by multiplying the prior distributions with the likelihood function and is given as [7]:

$$p\left(\theta |y\right)=\frac{p(y|\theta )p(\theta )}{p(y)}\propto p\left(y|\theta \right)p\left(\theta \right).$$ (11)

This is known as the principle of Bayesian approach. Here, *p*(*y*) is the prior distribution and the likelihood *p*(*y*|*θ*) is given as [7]:

$$p\left(y|\theta \right)=\prod_{i=1}^{N_{obs}}p\left(y|\theta \right).$$

The likelihood function of Bayesian approach is the same as that of classical approach. Thus, from Eq (7), replacing *θ* with the unknown parameter *β* we have that:

$$p\left(y|\theta \right)=p\left(y|\beta ,\phi_{j}\right)=\prod_{i=1}^{N_{obs}}\Delta_{i}^{y_{i}}{\left(1-\Delta_{i}\right)}^{1-y_{i}}.$$

The commonly used and the simplest prior for logistic regression parameters is a multivariate normal distribution. That is [7, 16]:

$$\beta ∼N(\Lambda_{0},\Sigma_{0}^{2}).$$

Thus we have:

$$p\left(\beta \right)\propto \exp\left[\frac{-1}{2}{\left(\beta -\Lambda_{0}\right)}^{{}^{\prime }}\Sigma_{0}^{-1}\left(\beta -\Lambda_{0}\right)\right].$$

It follows from Eq (11) that *p*(*β*|*y*_*i*_) ∝ *p*(*y*_*i*_|*β*)*p*(*β*). Hence, the posterior distribution for a logistic regression (considering only fixed effects) is given as:

$$p\left(\beta |y_{i}\right)\propto \prod_{i=1}^{N_{obs}}\Delta_{i}^{y_{i}}{\left(1-\Delta_{i}\right)}^{1-y_{i}}\cdot \exp\left[\frac{-1}{2}{\left(\beta -\Lambda_{0}\right)}^{{}^{\prime }}\Sigma_{0}^{-1}\left(\beta -\Lambda_{0}\right)\right].$$

This posterior distribution has a complex form which looks complicated to directly sample from. It is even more complex and complicated when the random effects is included in the model.

$$\begin{array}{lll} p(\beta ,\sigma_{\phi }^{2}|y_{i}) & \propto & p(y|\beta ,\sigma_{\phi }^{2})\cdot p(\beta |\sigma_{\phi }^{2})\cdot p\left(\sigma_{\phi }^{2}\right)\\ p(\beta ,\sigma_{\phi }^{2}|y_{i}) & \propto & \prod_{i=1}^{N_{obs}}\Delta_{i}^{y_{i}}{\left(1-\Delta_{i}\right)}^{1-y_{i}}\cdot \text{exp}\left[\frac{-1}{2}{\left(\beta -\Lambda_{0}\right)}^{{}^{\prime }}\Sigma_{0}^{-1}\left(\beta -\Lambda_{0}\right)\right]\cdot {\left(\sigma_{\phi }^{2}\right)}^{\alpha -1}e^{-\beta \sigma_{\phi }^{2}}, \end{array}$$

where $\sigma_{\phi }^{2}∼Gamma\left(\alpha ,\beta \right)$ meaning $p\left(\sigma_{\phi }^{2}\right)=\frac{\beta^{\alpha }}{\Gamma (\alpha )}{\left(\sigma_{\phi }^{2}\right)}^{\alpha -1}e^{-\beta \sigma_{\phi }^{2}}$.

Because of the complex nature of the posterior, we used the Markov Chain Monte Carlo (MCMC) technique to simulation of the random numbers following the posterior distribution.

The MCMC method is one of the most used method that generates the estimates of *θ* (unknown parameters) from appropriate distribution and then corrects the values generated to have a better estimate of the desired posterior distribution, *p*(*θ*|*y*) [7, 9]. If the posterior *p*(*θ*|*y*) is from a distribution which is complex or difficult to manipulate by the researcher, MCMC techniques provide a good alternative to summarize the posterior distribution. When using the MCMC to generate a sample of *p*(*θ*|*y*), we have to check that the MCMC algorithm converges to the desired posterior distribution.

#### Non-informative and informative priors

The most important aspect in Bayesian approach is to set up a proper prior to include in the model. The following method is employed in the selection of prior for the Bayesian approach with non-informative and informative prior.

A vague (non-informative) prior that will not influence the posterior distribution is chosen by using a normal distribution with a large variance (*σ*^2^ = 1000) and mean (*μ*_*j*_ = 1).

$$\beta_{k}∼N\left(1,1000\right),$$ (12)

The variance *σ*^2^ is transformed to inverse variance *τ* = 0.001 before being introduced in the model. The vague prior for the random effect is set up to be a gamma distribution with *α* = 0.1 and *β* = 0.01.

For the computation of the informative prior distribution of the Bayesian multilevel logistic regression, each fixed effect parameter is consider as $\beta_{k\gamma_{0}}∼N\left(\mu_{k\gamma_{0}},\sigma_{k\gamma_{0}}^{2}\right)$ where the mean $\mu_{k\gamma_{0}}$ is the mean of the coefficient related to the covariate *k* for the year *γ*_0_ = 2014. $\mu_{k\gamma_{0}}$ is obtained by averaging only the values of significant estimates ${\hat{\beta }}_{k}$ of all the previous years *γ* = (2011, 2012 to 2013). That is:

$$\mu_{k\gamma_{0}}=\frac{1}{N_{\gamma }}\sum_{\gamma =1}^{N_{\gamma }}{\hat{\beta }}_{k\gamma },$$

where *N*_*γ*_ is the total number of previous years where *β*_*k*_ was significant and ${\hat{\beta }}_{k_{\gamma }}$ is the estimate of parameter for each year *γ* between 2011 and 2013 where *β*_*k*_ was significant.

To compute the estimate of the variance $\sigma_{k\gamma_{0}}^{2}$, a pooled variance is used.

$$\sigma_{k\gamma_{0}}^{2}=S_{k\gamma_{0}}^{2}=\frac{\sum_{\gamma =1}^{N_{\gamma }}\left(n_{k\gamma }-1\right)S_{k\gamma }^{2}}{\sum_{\gamma =1}^{N_{\gamma }}\left(n_{k\gamma }-1\right)}$$

where *n*_*kγ*_ (*γ* = 2011, ⋯, 2013) is the number of observations for the covariate *k* in the year *γ* and $S_{k\gamma }^{2}\;\left(\gamma =2011,\cdots ,2013\right)$ is the corresponding variance.

For situations where an estimate is not statistically significant for any previous years between 2011 and 2013 a non-informative prior is used meaning: $\beta_{k\gamma_{0}}∼N\left(1,1000\right)$ with $\mu_{k\gamma_{0}}\;=\;1$ and $\sigma_{k\gamma_{0}}^{2}=1000$

The prior of the random effect parameter of a multilevel regression model is considered as a gamma distribution with parameters $\alpha_{\gamma_{0}}$ and $\beta_{\gamma_{0}}$: $\phi_{\gamma_{0}}\sim Gamma(\alpha_{\gamma_{0}},\beta_{\gamma_{0}}$. To compute an informative $\alpha_{\gamma_{0}}$ and $\beta_{\gamma_{0}}$, the following formulae of a gamma distribution parameters are used:

$$\alpha_{\gamma_{0}}=\frac{{\hat{Z}}_{\gamma_{0}}^{2}}{S_{\gamma_{0}}^{2}}\;\text{and}\;\beta_{\gamma_{0}}=\frac{S_{\gamma_{0}}^{2}}{{\hat{Z}}_{\gamma_{0}}}.$$

With ${\hat{Z}}_{\gamma_{0}}$ being the mean and $S_{\gamma_{0}}^{2}$ the corresponding variance of the gamma distribution in 2014 model. The problem of obtaining informative values of $\alpha_{\gamma_{0}}$ and $\beta_{\gamma_{0}}$ is then simplified to finding averages of ${\hat{Z}}_{\gamma }$ and $S_{\gamma }^{2}$ for previous years (for *γ* = 2011, ⋯, 2013):

$${\hat{Z}}_{\gamma_{0}}=\frac{1}{N_{\gamma }}\sum_{i=1}^{N_{\gamma }}{\hat{Z}}_{\gamma }\;\text{and}\;\;S^{2}=\frac{1}{N_{\gamma }}\sum_{i=1}^{N_{\gamma }}S_{\gamma }^{2}.$$

It is very important to check the convergence of the MCMC algorithm used in the study because a problem of non convergence or auto-correlation in the MCMC sample can lead to wrong results. Appropriate diagnostics such as; the Gelman-Rubin convergence diagnostic test, monitoring the Markov Chain (MC) error, checking for autocorrelation and observing the trace plots, can be used.

#### The Gelman-Rubin convergence diagnostic

The Gelman-Rubin convergence diagnostic was created by Gelman and Rubin in 1992 [7]. This test is only used when multiple chains are generated simultaneously. The diagnostic test is applied by computing and comparing within-sample variability and between-sample variability.

The within-sample variability (*WSS*) is the mean of variance among each sample. The between-sample variance $\frac{BSS}{T^{{}^{\prime }}}$ is the variance of the generated posterior mean over all the samples, where *T*′ denote the number of iterations used for each chain. The test statistics for the diagnostic test can be obtained using [7]:

$$\hat{R}=\frac{\hat{V}}{WSS}=\frac{T^{{}^{\prime }}-1}{T^{{}^{\prime }}}+\frac{\frac{BSS}{T^{{}^{\prime }}}}{WSS}\frac{k+1}{k},$$

where *k* is the number of chains or samples generated and

$$\hat{V}=\frac{T^{{}^{\prime }}-1}{T^{{}^{\prime }}}WSS+\frac{\frac{BSS}{T^{{}^{\prime }}}}{T^{{}^{\prime }}}\frac{k+1}{k}$$

is the pooled posterior variance estimate. $\hat{R}\to 1$ as convergence is attained.

## Results and discussion

The purpose of this paper is to determine significant predictors of TB in South Africa, comparing the classical approach and the Bayesian approach. To achieve this, we set up a generalized linear mixed model for the two approaches. The final outcome is to find predictors of individual’s risks of having TB in South Africa in 2014. Various predictors including HIV status and socio-economic factors are included in the model. The predictors (excluding the reference with the coefficient associated) introduced in the model are: The intercept (*β*_0_), Gender: Female (*β*_1_), Race: Non Black African (*β*_2_), Area of residence: Rural (*β*_3_), HIV status: No (*β*_4_), Working for a wage: No (*β*_5_), Marital status: Single (*β*_6_), Marital status: Separated/divorce/widow (*β*_7_), Highest Education Level: Primary (*β*_8_), Highest Education Level: Secondary or above (*β*_9_), Age (*β*_10_), Province (*β*.*Prov*)

Going through the descriptive analysis, it appears that out of the 9 provinces, Eastern Cape followed by Northern Cape have the highest percentage of respondents with TB. Descriptive results show that the percentage of males living with TB is greater than that of females. Also, areas of residence have equal proportion of respondents with TB.

In addition, people living with HIV have a higher prevalence of TB compared to those without HIV. People from the black community have a higher proportion of TB compared to others. Considering marital status, separated, divorced or widowed respondents have the highest prevalence, followed by the singles and married. Looking at the level of highest education attainment, results indicate that people with no education have a higher percentage of TB, followed by those with primary education level and those who have secondary school education level or more.

The classical and Bayesian multivariate generalized linear mixed models are fitted using the same predictors: The response variable *y* is TB or severe cough with blood (being the reference category). To test the significance of individual predictors, we used 95% Confidence Interval (CI) for classical statistics and 95% Credible Interval (Cred.I) for Bayesian approach. If these interval contains the number zero (0) we said that the parameter (estimate of the mean Beta) is not significant otherwise it is significant.

### Classical inference

The glmer function in R is used to fit the classical model. The result is given in Table 1 on Page 9.

**Table 1:** Model summary for the year 2014.

Parameters	Classical approach			Bayesian non-informative			Bayesian informative		
	Estimate	95%C.I		Estimate	95%Cred.I		Estimate	95%Cred.I	
		2.5%	97.5%		2.5%	97.5%		2.5%	97.5%
*β*_0_	-5.55*	-6.01	-5.08	-4.02*	-5.71	-2.44	-4.22*	-7.51	-1.38
*β*_1_	-0.42*	-0.43	-0.4	-0.51*	-0.82	-0.2	-0.51*	-0.81	-0.21
*β*_2_	-0.53*	-0.55	-0.51	-0.42	-0.91	0.05	-0.41	-0.90	0.05
*β*_3_	-0.13*	-0.15	-0.12	-0.14	-0.50	0.22	-0.11	-0.48	0.25
*β*_4_	-1.52*	-1.54	-1.5	-1.61*	-2.02	-1.18	-1.60*	-2.00	-1.17
*β*_5_	0.66*	0.64	0.67	0.57*	0.22	0.95	0.58*	0.22	0.95
*β*_6_	0.07*	0.05	0.09	0.12	-0.29	0.49	0.10	-0.28	0.48
*β*_7_	0.07*	0.05	0.09	0.16	-0.30	0.61	0.16	-0.30	0.61
*β*_8_	-0.14*	-0.16	-0.11	-0.21	-0.73	0.32	-0.22	-0.74	0.32
*β*_9_	-0.25*	-0.27	-0.22	-0.29	-0.80	0.24	-0.29	-0.81	0.26
*β*_10_	0.02*	0.02	0.02	0.02*	0.01	0.03	0.019*	0.01	0.03

The values with * are the significant values.

C.I = Confidence Interval and Cred.I = Credible Interval

Findings from this model reveal that all the variables used in the model are significant predictors of the risk of TB at 95% significant level or above. Considering gender, it appears that females are 34% (*OR* = 0.657) less likely to suffer TB compared to males. Also, results indicate that people from other population groups are 41% (*OR* = 0.589) less likely to suffer TB compared to black Africans.

Furthermore, findings reveal that people living in the rural areas are 12% (*OR* = 0.878) less likely to have TB compared to those living in the urban areas. Concerning the risk of coinfection TB and HIV, results show that people who are HIV negative are 78% (*OR* = 0.219) less likely to suffer TB compared to those living with HIV. Likewise, results illustrate that people unemployed are 1.94 times more at risk of having TB compared to those working for a wage, commission or salary.

Additionally, results about marital status highlighted that people who never got married are 1.07 times more likely to have TB as compared to those living with someone as husband and wife. For those who once got married (either divorced, widow or separated), findings led to an *OR* = 1.93 which indicates that, they are 1.93 times more likely to suffer TB compared to those living together as husband and wife. Furthermore, findings indicate that people whose highest education is primary school are less likely have TB compared than those who are not educated (*OR* = 0.869). Also, people whose highest education is secondary school or higher are 22% (*OR* = 0.779) less likely to have TB compared to those who are not educated. For age of respondents, result gives an *OR* = 1.022 indicating that the risk of TB increases 1.022 times as they grow a year older.

### Bayesian inference

For the Bayesian approach, WinBUGS software is used to fit the model. WinBUGS is a free software that is managed by Biostatistics unit of Medical Research Council (MRC) in the United Kingdom (UK). The same covariates as in Classical model are included in the Bayesian model. Two Bayesian models are fitted in this paper: Bayesian with vague or non-informative prior and Bayesian with informative prior.

#### Bayesian model with non-informative prior

The results for the model with non-informative prior are given in Table 1 on page 9. Findings show that the result of Bayesian with vague prior (data 2014) is very similar to that of classical statistics, which is in line with the theory. Indeed, the theory indicates that non-informative priors should not have effect on the posterior. The important part is that the credible interval for Bayesian statistics is very different from the confident interval for classical statistics. Indeed, the credible interval is more robust than the confidence interval which is highly affected (quality) by the sample size. That is why some variables significant in frequentist (classical statistics) are not in Bayesian inference with non-informative.

Considering the credible interval, results of non-informative prior Bayesian model indicate that variables; “Gender”, “HIV”, “Working for Wage” and “Age”, are the only significant predictors of the risk of having TB. While “Race”, “Area of residence”, “Marital status” and “level of education” are not significant determinants of TB in South Africa. Regarding the effects of gender on the risk of TB, findings highlight that females are 40% (*OR* = 0.6) less likely to have TB compared to males. Results also show that people that are HIV negative are 80.01% (*OR* = 0.20) less at risk of TB than those living with HIV. Furthermore, unemployed people are 1.77 times more at risk of having TB than others while as age increases by a year, the risk of having TB also increases by 1.02.

A Bayesian regression with vague prior is implemented also for 2011, 2012 and 2013 data and results are presented in Table 2. Results indicate that all significant predictors in 2013 were also significant predictors in 2012 and 2011. These predictors are gender, race, HIV status and education level. This indicates a certain pattern and consistency over the previous years. Indeed, males, black African, HIV positive and people with no education level appear to be consistently at high risk of having TB during the entire period 2011–2013. In addition, past data (2011–2013) analyses show some peculiarities such as unemployed and single people being at higher risk of TB than others in 2012 and unemployed and people living in rural areas being significantly at high risk of TB in 2011. Using all these information from past data analyses (2011–2013), informative priors were set up according to the procedure explained in the methodology and included in Table 2.

**Table 2:** Model summary for Bayesian approach with non-informative for the year 2011 to 2013 with the computed priors.

Parameters	2013 Output			2012 Output			2011 Output			Informative	
	Estimate	CI		Estimate	CI		Estimate	CI		Prior	
		97.5%	2.5%		97.5%	2.5%		97.5%	2.5%	mean	Var.
*β*_0_	-1.75	2.24	-44.9	-1.53	1.99	-30.6	-0.71	0.69	-2.09	1	1000
*β*_1_	-0.64*	-0.33	-0.95	-1.06*	-0.77	-1.36	-0.98*	-0.72	-1.24	-0.89	0.022
*β*_2_	-0.82*	-0.27	-1.42	-0.83*	-0.33	-1.38	-0.78*	-0.31	-1.28	-0.81	0.072
*β*_3_	0.05	0.40	-0.29	0.20	0.55	-0.15	0.48*	0.79	0.18	0.48	0.026
*β*_4_	-1.69*	-1.33	-2.05	- 1.78*	-1.39	-2.14	- 1.46*	-1.1	-1.88	-1.64	0.034
*β*_5_	0.33	0.72	-0.03	0.71*	1.09	0.35	0.82*	1.19	0.47	0.77	0.035
*β*_6_	0.25	0.64	-0.14	0.40*	0.78	0.03	-0.02	0.32	-0.35	0.40	0.036
*β*_7_	0.31	0.75	-0.14	-0.21	0.25	-0.70	-0.08	0.30	-0.47	1	1000
*β*_8_	0.03	0.53	-0.45	-0.03	0.45	-0.49	-0.22	0.16	-0.59	1	1000
*β*_9_	- 0.57*	-0.03	-1.10	-0.63*	-0.12	-1.12	- 0.58*	-0.18	-0.98	-0.59	0.059
*β*_10_	-0.01	9.5*e*^−4^	-0.02	-0.004	0.01	-0.02	-0.004	0.01	-0.01	1	1000
*τ*.Prov	50.05*	215.0	5.0*e*^−4^	16.11*	76.93	0.001	6.83*	19.63	1.35	*α* = 0.44	*β* = 55.20

values with * are the significant values.

**Fig 1:**
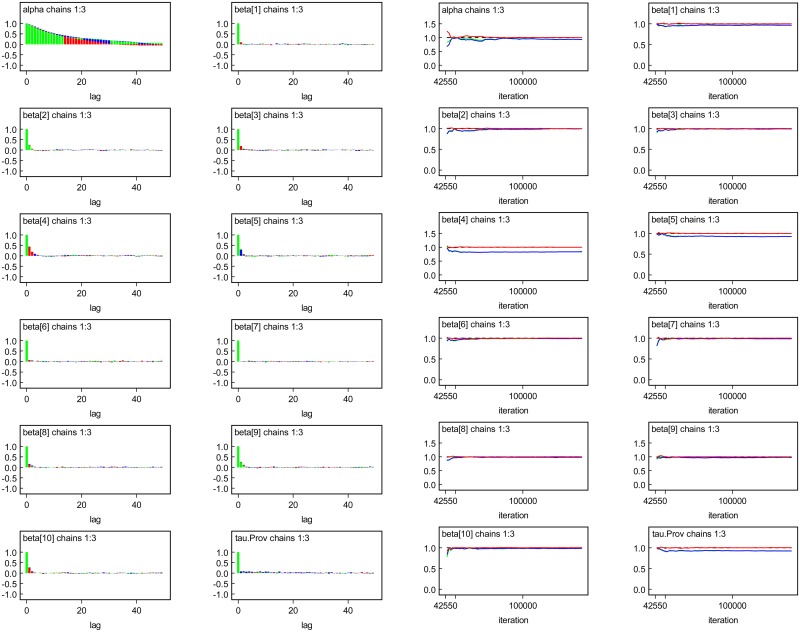
WinBUGS’ output of autocorrelation.

Once the results of the model are computed, it is important to check for the convergence of Markov Chain Monte Carlo. Fig 1 illustrates the convergence of the Bayesian with informative prior using the Gelman-Rubin Convergence Diagnostic test. The algorithm converged after 100, 000 iterations. To remove the autocorrelation and burning periods, a lag of 20 was considered and the first 35, 000 iterations removed. The output of Gelman-Rubin convergence diagnostic test displays the red lines representing the $\hat{R}$. The graph shows that all the $\hat{R}\to 1$. Also, the blue and green lines which represent the within sample variance and the pooled posterior variance, are stationary. Thus, the Gelman-Rubin Convergence Diagnostic test suggests that the algorithm converges.

#### Bayesian approach with informative prior

Most of the studies found in literature make use of non-informative prior in Bayesian inference. This is due to difficulties of availability of scientifically solid prior information about the unknown parameters. In this study, previous dataset for the year 2011 to 2013 were available and used to set up informative priors for the 2014 model. Table 2 gives the result of the informative priors. Bayesian approach with informative prior is conducted using the same model as that of the non-informative and classical models detailed in previous sections. The result of the Bayesian model with informative priors is given in Table 1.

The third model scrutinized in this paper consists of a Bayesian approach with informative prior. Results of this model displayed in Table 1 is almost identical to the results obtained for the Bayesian model with non-informative priors. This result indicates that the prior information are not strong enough or they are very close to the 2014 data. Results of the Bayesian with informative prior show that “Gender”, “HIV”, “employment” and “Age”, are the most significant predictors of the risk of having TB in South Africa while “Race”, “Area of residence”, “Marital status” and “level of education” are not significant determinants. The details suggest that females are 40% less at risk of having TB compared to males. Also, HIV negative respondents are 80.20% less at risk of TB compared to those who are HIV positive. In addition, those who are not working are 1.79 times more at risk of having TB compared to others who do. The result also shows that an increase in age increases the risk of having TB by 1.02.

### Discussion

At the end of this study, the results showed no significant difference between Bayesian with informative prior and Bayesian with non informative prior which could be due to the covariates utilized in this model or to the strength of the likelihood. However, Bayesian with informative prior is from definition better than the non informative prior because it includes scientifically based information in the model and the contribution of an informative prior vary from one model to another. On the other hand, the results from Bayesian approach are different from that of classical statistics, even Bayesian with vague priors produces results different from that of classical statistics.

Findings from Bayesian inferences and classical models are difficult to compare, the reason been that they are two fundamentally different techniques with different tools for decision making, *P*−value or confidence interval for one and credible interval for the other. However, when both techniques provide different results, findings from the Bayesian model are given preference because the technique is more robust and precise than the traditional (classical) statistics. Bayesian approach is usually criticized based on the choice of prior included in the model. Indeed, a wrong prior can include misleading information in the model which may result in misleading findings while a good prior will strengthen the quality of outputs. To avoid any doubt, two situation were considered in this study: a vague or non-informative prior with no real effect on the posterior and an informative prior set up using rigorous scientific steps. Findings of this study suggest that decision makers should give more credit to Bayesian model with informative priors than that of Bayesian with vague (non-informative) or classical statistics techniques. In conclusion, this study clearly advocates for the use of Bayesian findings compared to non Bayesian techniques.

Bayesian with informative and non-informative priors provided very close results. But the output of the informative prior is considered to be more precise and robust, compared to that of non-informative Bayesian model and classical model because of the presence of previous scientifically solid knowledge in the model. The use of informative priors (scientifically based evidence) or the addition of supplementary informative in the model will theoretically decrease the variance of the model and lead to a better model. It is based on the theoretical definition of informative prior (in addition to the result of our analysis which confirmed it), we concluded that Bayesian methods provide more precise and powerful result.

## Conclusion

Findings of the final model of this paper reveal that males, HIV positive, unemployed and older people are more at risk of TB than the others. Results also suggest that factors such as Race, Area of residence, Marital Status and Education Attainment are not statistically significant in the study of TB in South Africa.

One of the key discoveries of this paper is that Bayesian approach helps in selecting the more significant factors related to the risk of TB in South Africa, as compared to the classical approach that suggested that all the factors are significant. This paper suggests that to reduce the risk of TB in South Africa, attention should be given to old men, who are unemployed and HIV positive.
